# Urbanization Inequalities on the Incidence of Intraocular Cancers in Iran: An 11-Year Nationwide Retrospective Ecological Study

**DOI:** 10.18502/jovr.v20.16500

**Published:** 2026-01-12

**Authors:** Sare Safi, Zahra Khorrami, Mohadese Ahmadzade, Mohammad Esmaeil Akbari, Hamidreza Rouientan, Seyed Mohammadmehdi Moshtaghion, Mohammad Abolhosseini, Mozhgan Rezaei Kanavi, Saeed Karimi

**Affiliations:** ^1^Ophthalmic Epidemiology Research Center, Research Institute for Ophthalmology and Vision Science, Shahid Beheshti University of Medical Sciences, Tehran, Iran; ^2^Department of Optometry, Faculty of Rehabilitation, Shahid Beheshti University of Medical Sciences, Tehran, Iran; ^3^Ophthalmic Research Center, Research Institute for Ophthalmology and Vision Science, Shahid Beheshti University of Medical Sciences, Tehran, Iran; ^4^Cancer Research Center, Shahid Beheshti University of Medical Sciences, Tehran, Iran; ^5^Department of Regeneration and Cell Therapy, Andalusian Molecular Biology and Regenerative Medicine Centre (CABIMER), Seville, Spain; ^6^Ocular Tissue Engineering Research Center, Research Institute for Ophthalmology and Vision Science, Shahid Beheshti University of Medical Sciences, Tehran, Iran; ^7^The first and second authors equally contributed to this manuscript

**Keywords:** Incidence, Intraocular Cancers, Iran, Urbanization

## Abstract

**Purpose:**

To report the relationship between urbanization and the incidence of intraocular cancers in Iran.

**Methods:**

In this retrospective ecological study, data were extracted from the Statistical Center of Iran, the Meteorological Organization, and the Iranian National Population-based Cancer Registry (INPCR, 2006–2016). The urbanization index (UI) was calculated using principal component analysis, and weighted multiple linear regression was used to assess the relationship between the UI and the age-standardized incidence rate (ASIR) of intraocular cancers.

**Results:**

Six hundred and fifty-four intraocular cancer cases with a gender ratio (male-to-female) of 1:13 were recorded. The mean total 11-year ASIR of intraocular cancers was 0.093 per 100,000 population. The mean ASIR showed a decrease of 0.051 per 100,000 from 2006 to 2016 (*P* = 0.022). Retinoblastoma (66.1%) and uveal melanoma (24.6%) were the most commonly diagnosed intraocular cancers, and their ASIR declined between 2006 and 2016. Once adjusted for environmental pollutants, an increase in the area-based UI was significantly associated with lower total ASIR of intraocular cancers in males ( β = –0.14).

**Conclusion:**

Urbanization was associated with a reduction in ASIR of intraocular cancers, which can be attributed to improved access to eye care facilities for timely diagnosis. However, given that the INPCR cases were verified only by histology, future research should be conducted on hospital-based registries using multiple data sources to address confounders and exposure factors and prevent underestimation of incidence rates.

##  INTRODUCTION 

Intraocular cancers are rare but accounted for 84% of all eye cancers in the United Kingdom between 2016 and 2018.^[1,2]^ Intraocular cancers are among the most challenging cases managed by ophthalmologists.^[3,4,5,6]^ These cancers may lead to vision impairment, facial disfigurement, as well as psychological and learning problems. By 2015, the age- and sex-adjusted incidence rate of intraocular tumors in the United States was 727.5 per million.^[7]^ The most prevalent intraocular cancers are retinoblastoma in pediatrics and uveal melanoma in adults.^[8,9]^ Retinoblastoma accounts for 4% of all pediatric malignancies, with a survival rate of 50% in developing countries and 95% in developed countries.^[8]^

Urbanization is a multifactorial process comprising environmental, social, and economic domains that can drive economic growth, poverty reduction, and human development.^[10]^ However, it can impose negative impacts on people's health by increasing air pollution, low physical activity, obesity, and hypertension.^[11,12,13]^ Lifestyle changes resulting from urbanization may significantly increase the incidence of malignant cancers in developing countries.^[14,15]^ Some studies have reported a possible relationship between the incidence of intraocular cancers and various aspects of urbanization.^[16,17,18]^ Focusing on environmental factors, social behavior, and socioeconomic status, an Australian study (2021) found a higher incidence of intraocular melanoma in rural areas.^[16]^ Furthermore, the level of parental education in highly urbanized areas in China was reported to be positively correlated with their knowledge of screening for retinoblastoma.^[18]^

Globally, the proportion of the urban population has increased over the past few decades.^[19]^ Iran has experienced rapid urbanization and a rise in metropolitan populations in recent years.^[20,21]^ It was estimated that the proportion of people living in urban areas has more than doubled from 1950 to 2010, and it is projected that Iran's urbanization rate will increase up to 85% by 2050.^[22]^ Research supports the increased incidence of breast, colorectal, prostate, lung, and pancreatic cancers in more urbanized areas in Iran.^[23,24]^ Nevertheless, this relationship has not been investigated with regard to ocular cancers in Iran. Since intraocular cancers are the most life-threatening ocular disorders, this study was conducted to examine the relationship between the urbanization index (UI) and the incidence of intraocular cancers in Iran.

##  METHODS

### Study Design and Area

The present retrospective ecological study was conducted across all 31 provinces of Iran. From 2006 to 2016, the Iranian population increased from 70,495,782 to 79,926,270.^[25]^ Development across the provinces is significantly different due to their varied population densities, socioeconomic conditions, and other environmental pollution factors. The data for the area-specific analysis were obtained from the Statistical Center of Iran and the Iran Meteorological Organization.^[26]^ The prevalence of smoking was obtained from the 2016 STEPS Non-Communicable Disease Risk Factors Survey report.^[27]^

Ethics approval (IR.SBMU.ORC.REC.1401.010) was obtained from the Ethics Committee of the Ophthalmic Research Center, affiliated with Shahid Beheshti University of Medical Sciences, Tehran, Iran.

### Incidence of Intraocular Cancers

Information on patients with intraocular cancers diagnosed between 2006 and 2016 was obtained from the Iranian National Population-based Cancer Registry (INPCR).^[28]^ This registry was established about four decades ago by the Ministry of Health and Medical Education in collaboration with medical universities. All intraocular cancer cases were included based on the identification codes provided in the third edition of the International Classification of Diseases for Oncology (ICD-O-3). All cases were histopathologically confirmed and coded according to the ICD-O-3. Only cases of verified histology were enrolled in our study. Topographic codes C69.2 (retina), C69.3 (choroid), and C69.4 (ciliary body) were used to identify intraocular cancers. All cases were coded according to the morphology codes in the ICD-O-3: codes 9510-9513 for retinoblastoma and 8720 and 8770-8774 for melanomas. Other intraocular cancers were coded as follows: 8000 for unspecified cases; 8010, 8070, 8090, 8140, 8240, 8510, and 8525 for carcinoma and adenocarcinoma; 8890 and 8900 for sarcoma; 9501 and 9520 for neurologic cases; and 9590, 9663, and 9699 for lymphoma. Intraocular cancer cases were classified into 17 age groups by topography to serve as the basis for age standardization. The data were standardized for the whole country by the direct method using the World Standard Population (2004).^[29]^ In addition, the age-standardized incidence rate (ASIR) was analyzed by gender in each province, standardized using the direct method based on the 2016 Iranian population census. Due to variations in the ASIR of intraocular cancers among provinces, the mean ASIR for each province was used in the analysis.

### Urbanization Index (UI)

Principal component analysis was used to create the UI, based on 22 indicators, for 31 provinces in 2006 and 2016.^[30]^ We selected indicators based on *a previous* knowledge and available data for the provinces of Iran [Supplementary 1]. The indicators included Gross Domestic Product (GDP), province's portion of the country's GDP, inflation rate, average household size, average income of urban households, average consumption costs in urban households, economic partnership rate, unemployment rate, urbanization rate, telephone lines (fixed-line and mobile), the modified drinking water quality index, population covered by the municipal sewage system, life expectancy, literacy rate, density of physicians, density of emergency facilities, density of health centers, density of active hospital beds, human development index, Gini index, and the human poverty index.

### Other Explanatory Variables

The environmental quality of each province was extracted based on the measurement of seven main parameters: mean particulate matters (‎PM ${}_{2.5}$ and PM ${}_{10}$ ), ‎Ozone (O ${}_{3}$ ), carbon compounds (CO, CO ${}_{2}$ ),^[31]^ nitrogen compounds (NO, NO ${}_{2}$ ), sulfur dioxide (SO ${}_{2}$ ), ultraviolet radiation,^[32]^ and averaged days of exposure to dust per year. The parameters were log-transformed, and the environmental quality of each province was calculated with the K-means cluster.^[33]^ Thereafter, the provinces were categorized into areas with higher and lower pollution; accordingly, 20 provinces (64.5%) were associated with higher environmental pollution.

### Statistical Analysis

The ASIR of intraocular cancer in each province was presented as the mean by gender and topography. Except for retinoblastoma and uveal melanoma, other intraocular cancers recorded in the INPCR were rare. As a result, statistical analysis was performed on these two cancers, as well as on total intraocular cancers. The variability in the ASIR across provinces was presented as standard deviation and coefficient of variation (CV%). Given that the accuracy of the estimation of the incidence rate depends on the size of the population and a higher UI in larger cities with more population in Iran, a weighted multiple-linear regression model was used to assess the relationship between UI and ASIR.^[34,35]^ The size of the population of each province was included as a weight in the model. The results of the models were presented as beta coefficients with 95% weighted confidence intervals (95% WCI) for two scenarios across three models (Models A, B, and C). The first scenario represented the relationship between ASIR and the UI in 2016. The second scenario denoted the relationship between ASIR and the change in UI between 2006 and 2016. Three linear models were constructed for each scenario. The relationship between ASIR and UI was estimated in Model A. The level of environmental pollution was adjusted in Model B. The prevalence of smoking was adjusted for the relationship between uveal melanoma ASIR and the UI in Model C. Pearson correlation analysis was performed to analyze the correlation between the UI and the mean ASIR in all provinces. Statistical analyses were performed using Stata V.14 (Stata, College Station, Texas, USA), and ArcGIS V.10.8.2 (Esri, Redlands, California, USA) was used for data visualization.

**Table 1:** Number, percentage, crude rate, and age-standardized incidence rate (per 100,000) of intraocular cancers in males and females in Iran during the period 2006–2016

	**Number **	**Percentage (%) **	**Crude rate**	**ASIR**
Male				
Retinoblastoma	237	68.1	0.486	0.212
Melanoma	71	20.4	0.116	0.079
Others ${}^{*}$	40	11.5	0.049	0.073
Total	348	100	0.429	0.331
Female				
Retinoblastoma	195	63.7	0.415	0.172
Melanoma	90	29.4	0.149	0.088
Others ${}^{*}$	21	6.9	0.027	0.074
Total	306	100	0.388	0.296
*Other intraocular cancers include carcinoma and adenocarcinoma (*n* = 22), lymphoma (*n* = 4), sarcoma (*n* = 3), neurologic (*n* = 2), and unclassified malignant tumors (*n* = 30); ASIR, age standardized incidence rate.				

**Table 2:** Regional variability in the incidence of intraocular cancers across different provinces of Iran during the period 2006–2016

	**Male**			**Female**		
	**Mean ASIR ± SD**	**Min–Max**	**CV (%)**	**Mean ASIR ± SD **	**Min–Max**	**CV (%)**
Retinoblastoma	0.212 ± 0.15	0.0–0.56	70.75	0.172 ± 0.14	0.0–0.44	81.39
Melanoma	0.078 ± 0.06	0.0–0.25	76.92	0.087 ± 0.07	0.0–0.31	80.46
Others ${}^{*}$	0.028 ± 0.04	0.0–0.15	142.86	0.026 ± 0.05	0.0–0.16	192.31
Total	0.331 ± 0.19	0.0–0.76	57.40	0.296 ± 0.18	0.0–0.64	60.81
*Other intraocular cancers include carcinoma and adenocarcinoma (*n* = 22), lymphoma (*n* = 4), sarcoma (*n* = 3), neurologic (*n* = 2), and unclassified malignant tumors (*n* = 30); ASIR, age-standardized incidence rate; CV, coefficient of variation of ASIR between different provinces; Max, maximum; Min, minimum.						

**Table 3:** Relationship between the urbanization index in 2016 and the mean ASIR of intraocular cancers in Iran

	**Model**	**Male**			**Female**		
		*** β ***	**95% CI**	***P*** **-value**	*** β ***	**95% CI**	***P*** **-value**
Retinoblastoma	A	*–*0.034	–0.094 to 0.025	0.245	–0.016	–0.073 to 0.041	0.566
	B	–0.044	–0.106 to 0.017	0.151	–0.015	–0.076 to 0.045	0.604
Melanoma	A	–0.033	–0.055 to 010	0.006	–0.009	–0.038 to 0.019	0.484
	B	–0.029	–0.052 to 0.006	0.015	–0.002	–0.031 to 0.026	0.863
	C	–0.028	–0.053 to 0.003	0.031	–0.008	–0.042 to 0.026	0.630
Total	A	–0.085	–0.157 to 0.014	0.020	–0.019	–0.091 to 0.052	0.581
	B	–0.092	–0.167 to 0.017	0.018	–0.018	–0.094 to 0.058	0.629
Model A, crude coefficient; Model B, adjusted for environmental pollution; Model C, adjusted for environmental pollution and prevalence of smoking; CI, confidence interval; ASIR, age-standardized incidence rate per 100,000 population; Statistically significant was defined as *P* < 0.05; 20 provinces (64.5%) had higher environmental pollution, according to K-means cluster analysis in model B. One unit increase in the urbanization index was associated with a 1-standard-deviation decline in the mean ASIR. Model C was not applicable to retinoblastoma and total intraocular cancers.							

**Table 4:** Relationship between changes in urbanization index (2006–2016) and the mean ASIR of intraocular cancers in Iran

	**Model**	**Male**			**Female**		
		*** β ***	**95% CI**	***P*** **-value**	*** β ***	**95% CI**	***P*** **-value**
Retinoblastoma	A	–0.06	–0.147 to 0.025	0.160	–0.087	–0.164 to 0.011	0.027
	B	–0.066	–0.153 to 0.21	0.133	–0.088	–0.167 to 0.008	0.031
Melanoma	A	–0.045	–0.078 to 0.011	0.010	0.001	–0.043 to 0.042	0.975
	B	–0.042	–0.075 to 0.009	0.014	0.004	–0.036 to 0.045	0.826
	C	–0.038	–0.072 to 0.004	0.029	0.002	–0.04 to 0.045	0.907
Total	A	–0.140	–0.241 to 0.038	0.009	–0.072	–0.173 to 0.029	0.155
	B	–0.141	–0.246 to 0.037	0.010	–0.071	–0.175 to 0.032	0.170
Model A, crude coefficient; Model B, adjusted for environmental pollution; Model C, adjusted for environmental pollution and prevalence of smoking; ASIR, age-standardized incidence rate per 100,000 population; Statistical significance was defined as *P* < 0.05; Model C was not applicable to retinoblastoma and total intraocular cancers.							

**Figure 1:**
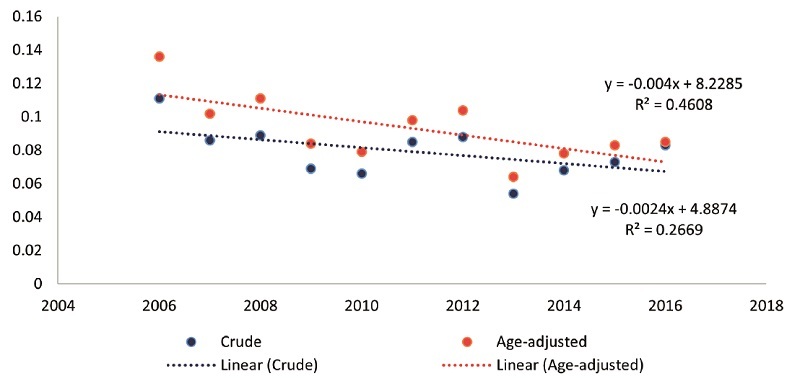
Annual ASIR of total intraocular cancers from 2006 to 2016 in Iran. The red and blue lines represent the decreasing ASIR and crude incidence rates per 100,000 population, respectively. ASIR, age-standardized incidence rate.

**Figure 2:**
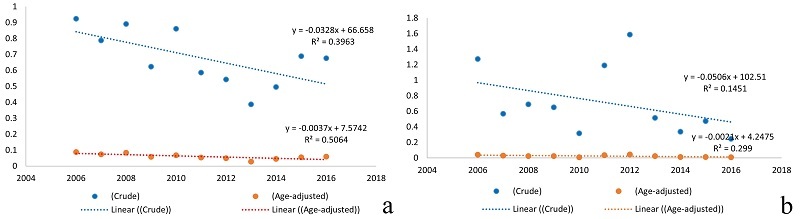
Annual ASIR of (a) retinoblastoma and (b) uveal melanoma from 2006 to 2016 in Iran. The red and blue lines represent the decreasing ASIR and crude incidence rates per 100,000 population, respectively. ASIR, age-standardized incidence rate.

**Figure 3:**
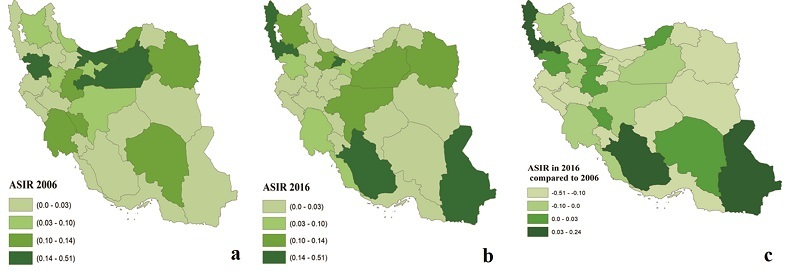
ASIR of total intraocular cancers in (a) 2006, (b) 2016, and (c) their change across different provinces of Iran. ASIR, age-standardized incidence rate.

**Figure 4:**
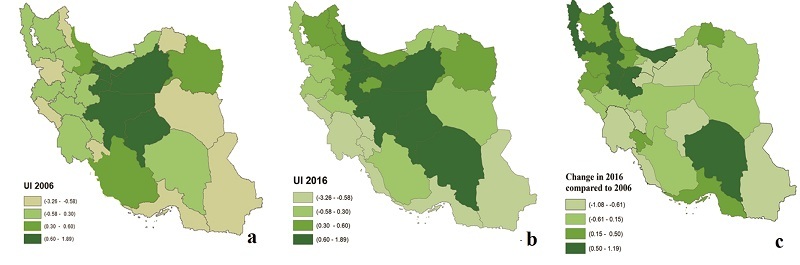
Urbanization index in (a) 2006, (b) 2016, and (c) its change across different provinces of Iran.

**Figure 5:**
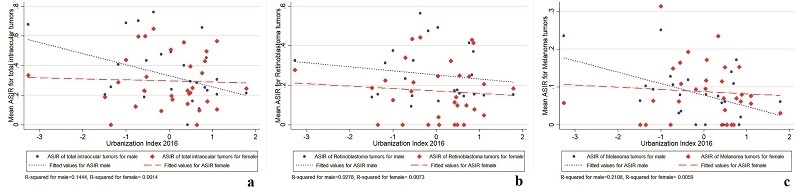
Provincial-level correlation of urbanization index and age-standardized incidence rate of (a) total intraocular cancers, (b) retinoblastoma, and (c) uveal melanoma in 2016 in Iran (red line: female; blue line: male).

##  RESULTS

### Incidence of Intraocular Cancers

A total of 654 intraocular cancer cases were recorded in 31 provinces of Iran from 2006 to 2016. The gender ratio (male-to-female) for all intraocular cancer cases was 1:13. The highest number of intraocular cancer cases (*n* = 78, 11.9%) was identified in 2006. The mean 11-year ASIR of total intraocular cancers was 0.093 per 100,000 population. The ASIR of intraocular cancers decreased from 0.136 to 0.085 per 100,000 population between 2006 and 2016. The decreasing trend of the overall average annual crude incidence rates (*P* = 0.099) and ASIR (*P* = 0.022) for intraocular cancers is presented in Figure 1 and Table 1.

The highest number of intraocular cancers was related to retinoblastoma (*n* = 432, 66.1%), followed by uveal melanoma (*n* = 161, 24.6%), and others (*n* = 61, 9.3%). The mean 11-year ASIRs of retinoblastoma and uveal melanoma were 0.172 and 0.088 per 100,000 population, respectively. The distribution of ASIR of retinoblastoma was moderately higher in males than in females (*P* = 0.059) [Table 1]. Figure 2 shows the declining ASIR of retinoblastoma and uveal melanoma from 2006 to 2016 (*P* = 0.014 for retinoblastoma and *P* = 0.082 for uveal melanoma).

We identified an unequal distribution of ASIR for intraocular cancers across different provinces in Iran (CV = 52%). The highest increase in ASIR was detected in Fars (0.237 per 100,000 population), followed by West Azerbaijan (0.225 per 100,000 population), and Sistan and Baluchestan (0.147 per 100,000 population) from 2006 to 2016. Additionally, the ASIR changes of all intraocular cancers were higher in females (CV: 60.81%; Ilam = 0.0 vs. Golestan = 0.649) [Table 2 & Figure 3].

### Urbanization Index (UI) 

The UI ranged from –2.19 to 1.89 in 2006 and from –3.26 to 1.78 in 2016 across the 31 provinces. The border provinces (West, South, and Southeast) had the lowest UIs, whereas the central provinces marked the highest UIs in 2016. Eighteen provinces (58%) showed an increase in UI from 2006 to 2016 [Figure 4].

### Urbanization Inequality and Intraocular Cancers Incidence

The correlation of the UI in 2016 with the mean ASIR of total intraocular cancers and uveal melanoma was remarkably significant in males (total intraocular cancers: *r* = –0.38, *P* = 0.035; uveal melanoma: *r* = –0.46, *P* = 0.010) [Figure 5]. This inverse relationship was confirmed after adjusting for smoking prevalence and environmental pollution at the provincial level. The relationship between the UI in 2016 and the mean ASIR of intraocular cancers is shown in Table 3. The increase in the area-specific UI between 2006 and 2016 is significantly related to the lower ASIR of total intraocular cancers (* β* = –0.141), uveal melanoma in males (* β* = –0.045), and retinoblastoma in females (* β* = –0.087) [Table 4].

##  DISCUSSION

This study analyzed the national epidemiological trends in the incidence of intraocular cancers in relation to urbanization in Iran. The mean 11-year ASIR of total intraocular cancers was 0.093 per 100,000 in the current study. Our results showed a decreasing trend in the average annual crude incidence rates and the ASIR for intraocular cancers over an 11-year period (2006–2016). The distribution pattern of ASIR for intraocular cancers varied across regions of Iran. Our findings attribute the increase in the area-specific UI to a decline in the incidence of total intraocular cancers, with a specific decline in uveal melanoma in males and retinoblastoma in females.

Similar to our study, a population-based study in Olmsted County, Minnesota, US, reported a decreasing trend in the ASIR of intraocular cancers between 2006 and 2015.^[7]^ Nevertheless, the ASIR of intraocular cancers in that study was 0.95 per 100,000 (95% CI, 0.41 to 1.49) versus 0.093 per 100,000 in our study.^[7]^ This difference might be justified by the data source. In that study, the data were extracted from a database that included the medical records of almost all county residents, whereas the current study focused only on cases verified by histopathology. In our study, retinoblastomas had the highest prevalence of intraocular cancers (66%), consistent with another report from the Philippines (91%).^[3]^ The mean ASIR of retinoblastoma in South Africa over a 15-year period was 0.33 per 100,000,^[36]^ which was in line with our study results. In the current study, the ASIR measurements for retinoblastoma were higher in males, which may be explained by the preference of parents to ensure nonsurgical treatment modalities in their female children due to aesthetic concerns. Our results also showed that the registration rate for retinoblastoma declined significantly over the 11-year study period. In contrast, reports from Jordan (2006–2010),^[17]^ Pakistan (1998–2002),^[38]^ Canada (1992–2010),^[39]^ and the United States (1975–2004)^[40]^ suggest a stable trend over the investigated periods.

The discrepancy in trend patterns observed in our study versus other studies may be attributed to differences in data collection methodologies and the increasing implementation of globe-salvaging treatment methods, which became more common in retinoblastoma management over the study period at referral centers in Iran.^[37]^ These advances may have contributed to fewer registrations and, consequently, an underestimation of ASIR trends.

Uveal melanoma was the second most common intraocular cancer in the present study (24.6%). The mean ASIR of uveal melanoma over 11 years (0.088 per 100,000 population) was lower than that in Western countries. The ASIR of choroidal melanoma and iris melanoma in the Minnesota study was 0.71 per 100,000 (95% CI, 0.25 to 1.18) and 0.09 per 100,000 (95% CI, 0.0 to 0.27), respectively.^[7]^ In another study from Germany (2009–2015),^[41]^ the ASIR of uveal melanoma was 0.641 per 100,000 (95% CI, 0.62 to 0.66). In two Eastern states of Australia (2001–2013), the ASIR of uveal melanoma ranged from 0.46 to 0.61 per 100,000 per year.^[16]^ These discrepancies in the ASIR measurements may be attributed to the ethnic pigmentation of the skin and uvea of patients in our region, as these ethnicities can withstand the melanogenic effects of solar radiation.^[42,43,44]^ Similar to retinoblastoma cases that are managed with globe-salvaging therapies, patients with uveal melanoma who had undergone such treatments are not captured in our data. According to a recent longitudinal single-center study in Iran, this exclusion could account for as much as two-thirds of uveal melanoma cases,^[45]^ leading to a potential underestimation of ASIR.

Based on the present study, the ASIR of intraocular cancers varied significantly across regions and revealed an inverse correlation with the UI. The highest level of regional variability in the ASIR of total intraocular cancers was observed in females. Our study also showed an inverse relationship between the UI and the ASIR of retinoblastoma in females. Environmental factors such as parents' place of residence, father's exposure to chemical agents, and mother's exposure to viral infections before pregnancy are possible risk factors influencing regional variations in the ASIR of retinoblastoma.^[46]^ Our study also showed an inverse relationship between the UI and the ASIR of uveal melanoma in males. Uveal melanoma had the highest regional variability in males. Chalada et al demonstrated that rural areas have a higher ASIR of uveal melanoma than urban areas, suggesting that a multitude of factors, including chemical exposure, social behavior, and socioeconomic status, may contribute to this difference.^[16]^ The relationship between uveal melanoma and exposure to sunlight has been reported in different studies, including those conducted on Swedish construction workers,^[47]^ American fishermen and sailors,^[48]^ Australian and American farmers,^[49,50]^ and Canadian railway workers.^[51]^ Following urbanization, the proportion of employees engaged in primary industries (agriculture, forestry, animal husbandry, and fishing) has decreased dramatically.^[52]^ For instance, by 2011, only 34.8% of Chinese laborers worked in agriculture, compared with 60% in the 1960s.^[53]^ Similarly, the percentage of employment in agriculture in Iran decreased from 23% in 2006 to 18% in 2016.^[54]^ Thus, urbanization has reduced the proportion of sun-exposed occupations and the consequent risk of ultraviolet exposure. Furthermore, recent research suggests that having children increases the risk of developing uveal melanoma among women of childbearing age due to changes in hormone levels during pregnancy.^[55,56,57]^

To the best of our knowledge, the current study is the first national report on the relationship between UI and ASIR of intraocular cancers, based on the data from INPCR, in the Eastern Mediterranean Region. Providing data for all 31 provinces, the INPCR is one of the most reliable sources of cancer incidence data in Iran and has been utilized by several nationwide studies. In addition, the UI can help policymakers reach large-scale regional decisions.

The present study has some limitations. The lack of individual-level information due to the ecological nature of the study and, consequently, the “ecological fallacy” (an intrinsic limitation of ecological studies) that prevents making causal inferences are the main limitations of the study. However, urbanization is basically an ecological process that includes multiple environmental components. Moreover, our study lacks information about genetic predispositions and access to healthcare services among patients recorded in the INPCR database. Given that this information is usually obtained through family contact details or patient interviews, a further study can be designed to address this limitation. Furthermore, the exclusion of cases not verified by histology, such as those treated with intra-arterial chemotherapy or systemic chemotherapy, might have led to an underestimation of the incidence rates of intraocular cancers. In this regard, it is recommended to integrate multiple data sources, such as electronic health records, insurance databases, and laboratory systems, to apply the capture-recapture method and perform regular quality controls.^[58]^

In conclusion, Iran has seen an increasing trend of urbanization over a period of 11 years (2006–2016). This trend was associated with economic development and improved health systems that can affect both the incidence and management of diseases. We found an inverse relationship between increasing UI and the ASIR of intraocular cancers. The lower ASIR of intraocular cancers in more urbanized areas may be explained by increased accessibility to globe-salvaging therapies, which were not included in this study.Hence, future studies are recommended to use both hospital-based registries and INPCR to assess the relationship between ASIR of intraocular cancers and UI.Diagnosing intraocular cancers, especially retinoblastoma, is important for improving quality of life and productivity; therefore, future studies should explore the effects of ecological indicators and lifestyle risk factors on the incidence of intraocular cancers. This knowledge will help policymakers and assist in shifting protocols from tertiary to secondary and primary prevention levels, and facilitating individual-based registration via multiple data sources.

##  Financial Support and Sponsorship

None.

##  Conflicts of Interest

None.
